# Communal rearing induces high predatory capacity in a solitary wolf spider and its potential in pest control

**DOI:** 10.1002/ece3.10024

**Published:** 2023-04-18

**Authors:** Yaqi Peng, Fan Zhang, Die Hu, Dong Li, Yao Zhao, Yu Peng

**Affiliations:** ^1^ Hubei Key Laboratory of Regional Development and Environmental Response, Faculty of Resources and Environmental Science Hubei University Wuhan China; ^2^ State Key Laboratory of Biocatalysis and Enzyme Engineering, School of Life Sciences Hubei University Wuhan China

**Keywords:** biological control, communal rearing, foraging behavior, large‐scale breeding, spider

## Abstract

Behavioral plasticity is of great significance because it allows individuals to flexibly respond to variations in the ecological and social environment. To date, there is little published data on the topic of whether the early rearing experience of spiders influences their later foraging behavior. *Pardosa pseudoannulata* (Araneae: Lycosidae) is a solitary wolf spider, it is a major predator of pests such as *Nilaparvata lugens* in rice fields. In this study, we aim to develop a communal rearing protocol for spiders. We conducted a rearing study in the lab that one group of wolf spiders was reared communally and a second group was reared individually. We compared the survival rates and predatory capacity of *P. pseudoannulata* in both settings. Survival rates were similar overall. At forty‐five days, survival rates were below 40% for both groups. Raising spiders communally led to higher foraging levels. Across all tested time points, spiders reared communally hunted more fruit flies than those reared individually. Significant differences were found between the two rearing groups after hunting for seven and 10 min. Field experiment showed that release of communal‐reared spiders significantly reduced the pest *N. lugens* population. Our research provides reference for the large‐scale breeding of spiders and their application as biological control agents.

## Graphical abstract

*Pardosa pseudoannulata* is a solitary wolf spider and it is a major predator of pests such as *Nilaparvata lugens* in rice fields. The results showed that the survival rates of the communal‐reared and individual‐reared spiders were similar, but communal‐reared spiders were significantly more voracious. Field experiments further demonstrated that release of communal‐reared spiders could significantly reduce the number of the pest *Nilaparvata lugens*.

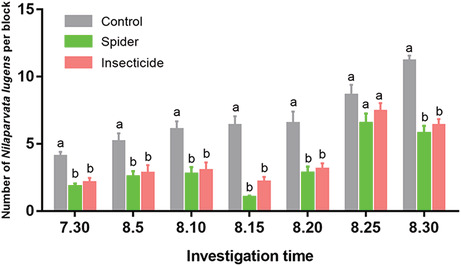

## 1 INTRODUCTION

Behavioral plasticity is of great significance because it allows individuals to reversibly respond to variations in the ecological and social environment to increase their fitness (Bretman et al., 2011). Early exposure to new ornamentations in fruit flies (Verzijden et al., 2015) or butterflies (Westerman et al., 2012) led to shifts in mate preferences in sexually mature older individuals. Many other studies have reported that prior experience influences mate choice in animals (Bailey & Zuk, 2008; Hebets, 2003; Meyer et al., 2019; Qvarnström et al., 2000).

The impact of the early social environment has been well studied in rodents (Branchi, 2009), in birds (Adkins‐Regan & Krakauer, 2000; Gersick et al., 2012), in fishes (Moretz et al., 2007; Sykes et al., 2018), and in insects (Mortensen & Ellis, 2018). Moreover, the social‐rearing environment has large effects on animal behavior (Adkins‐Regan & Krakauer, 2000; Toth et al., 2011; Tóth et al., 2008). Of the cichlid fish *Pelvicachromis taeniatus*, which has mutual mate choice, males and females reared in isolation were less likely to perform courtship behavior and showed less interest in potential mates than group‐reared fish (Hesse et al., 2016). A recent study reported that significant differences were identified in the nursing behavior and sucrose responsiveness between bees reared in vitro and those in their parental colony (Mortensen & Ellis, 2018). Compared with lab‐reared males, field‐reared wolf spider *S. ocreata* males showed fewer behavioral differences based on female state, suggesting that male mate preference may be influenced by rearing experience (Meyer et al., 2019). According to a recent study, field‐collected spiders prefer red flowers with UV fluorescence, while lab‐reared spiders prefer red without UV, suggesting that lab‐reared and field‐collected animals can respond differently to the same experimental treatments (Wiggins et al., 2018). In addition, many researches involving behavioral plasticity in spiders have been conducted concerning sexual selection, web construction, communication, and exploration behavior (Blamires, 2010; Gordon & Uetz, 2011; Hesselberg, 2015; Stoffer & Uetz, 2017; Wilder & Rypstra, 2008a).

Early life experiences are known to influence behavior later in life. Differences among individuals in prior experiences may contribute to individual differences in behavioral plasticity (Stamps, 2015). Environmental enrichment could improve foraging behavior in hatchery‐reared Atlantic salmon (Brown et al., 2003). Spiders are predatory and cannibalistic, and more than 50,000 spider species described to date (World Spider Catalog, 2023), only dozens have been found living in groups (Lubin & Bilde, 2007). To date, there is little published research on the issue that whether early rearing experience of solitary spiders would affect their later foraging behavior. *Pardosa pseudoannulata* (Araneae: Lycosidae) is a wandering wolf spider that has large populations in rice fields in Asia, and it is a major predator of pests such as *Nilaparvata lugens* in the rice field (Lv et al., 2022; Tang et al., 2022). The planthopper *N. lugens* (Stål) (Hemiptera: Delphacidae) is one of the most serious pests of rice in both temperate and tropical regions of east and south Asia, and causes serious yield losses of rice crops all over the world (Liu, Li, et al., 2020; Liu, Zhuang, et al., 2020; Riley et al., 1991).

Spiders are rarely communally‐reared for biological control and we aim to develop a communal rearing protocol in present study. We tested how the protocol affected the spider behavior compared to individually reared spiders that were reared under common laboratory conditions. We changed several variables in the communal rearing protocol (i.e., spider density and environmental enrichment). We hypothesize that a communal rearing protocol would affect spider behavior compared to solitary‐reared spiders. Specifically, in this study, the spider *P. pseudoannulata* were reared communally or individually. Then, the survival rates and predatory capacity of the communal‐reared and individual‐reared spider were compared. Finally, field experiment was conducted to test the communal‐reared spiders whether they were effective at suppressing pest *N. lugens* populations.

## 2 MATERIALS AND METHODS

### 2.1 Spiders

*P. pseudoannulata* subadults were collected in May 2018 from rice fields at Huazhong Agricultural University, Wuhan, China. The spiders were fed with adult fruit flies (*Drosophila melanogaster*) every other day. The fruit flies were cultured in a medium composed mainly of corn meal, sucrose, and yeast extract powder. Immediately upon emerging, the spiderlings climb onto the mother's abdomen and aggregate there for several days before dispersing (Zhao, 1993). After dispersing, they show cannibalistic behavior (Zhao, 1993). In this study, when the *P. pseudoannulata* subadults were mature, males and females were paired in cylindrical glass tubes (20 mm diameter, 100 mm high) and allowed to copulate. After copulation, pairs of the spiders were separated. The females were then reared individually in glass tubes and allowed to lay egg sacs. After the egg sacs were hatched, the second instar spiderlings were used in the subsequent experiments. The first instar of *P. pseudoannulata* spiderlings molt once within the egg sacs and emerge from the egg sacs as second instars (Zhao, 1993).

### 2.2 Rearing experiments

The second instar spiderlings were reared in two different ways. The first method of group rearing was developed in this study (Figure S1A). A round sponge (30 mm thick) was put on the bottom of a transparent plastic bottle with a 5‐L capacity (153 mm diameter, 228 mm high, 115 mm caliber). The sponge was soaked, and thirty rice seeds were planted in the wet sponge. A round feeding hole (15 mm diameter) for adding fruit flies was made in the bottle cap and was blocked with a wooden plug (Figure S1B and Figure S1C). For ventilation, fifty small holes (1 mm diameter) were made in the cap (Figure S1B).

When the rice seedlings grew to about 60 mm high, second instar spiderlings were moved into this rearing bottle (Figure S1A). In total, 691 second instar spiderlings were divided into eleven bottles, with each bottle containing approximately sixty spiderlings. The spiderlings in each bottle were from one clutch. The rearing bottles were kept at 26 ± 1°C and 70 ± 5% relative humidity under a 14 h light:10 h dark cycle. Every day, the spiderlings were fed approximately 150 CO_2_‐anesthetized fruit flies through the funnel (Figure S1C). The sponge at the bottom was kept wet. Survival was recorded every day. The developmental periods of the communal‐reared spiders were generally observed, and many of the surviving spiders were speculated to be in fourth instar. Since the developmental period of each communal‐reared spider could not be determined, the communal‐reared spiders that were in similar size with the individual‐reared spiders (45‐days old, in fourth instar) were used in the subsequent predatory capacity experiment.

For the second rearing method, second instar spiderlings were individually reared in cylindrical glass tubes (same type as above; Figure S1D). The sponge on the bottom of the tube was kept wet, and each glass tube was sealed with cotton gauze. Every other day, each spiderling was fed 5 (before 20‐days old) or 10 (after 20‐days old) CO_2_‐anesthetized fruit flies. In total, 113 second instar spiderlings from five different egg sacs were reared in this way, and survival was recorded every day. These five egg sacs were different from the clutches used in the communally reared group. The temperature and humidity were maintained at the same levels as described above. Fourth instar spiderlings (45‐days old) were used in the subsequent predatory capacity experiment.

### 2.3 Predatory capacity

The predatory capacity of the spiderlings from both rearing methods was compared. For the solitary group, thirty spiderlings (all in fourth instar) were randomly selected from the 113 glass tubes. For the communally reared group, ten bottles were randomly selected from the eleven bottles, and then three spiderlings (in similar size with the individual‐reared spiders) were selected from each bottle. In total, thirty spiderlings from each rearing setting were tested for predatory capacity. Each spiderling was transferred to a glass tube (with a wet sponge in the bottom), where they were starved for 5 days. After starvation, fifteen live fruit flies were transferred into each glass tube. Predation numbers were recorded within four, seven, and 10 min.

### 2.4 Field experiment

The field experiments were conducted during the August 2018 in the suburbs of Wuhan City, in Hubei Province, China. The rice fields were planted with the Chinese rice cultivar “Guangliangyouxiang66” seeds. Before the experiments, three non‐neighboring rice fields were chosen based on a similar number (100 per field) of the pest *N. lugens*. Extra *N. lugens* and other arthropods including the spider *P. pseudoannulata* were removed manually by insect suction vacuum. Each of the rice fields was in an area of 600 m^2^. The first rice field was set as the control field, no spider was released or insecticide was used throughout the field experiment. The second rice field was released evenly by 500 communal‐reared spiders (reared according to the methods described above) on 25th July, and no insecticide was applied. The third rice field was applied with the botanical insecticide veratrine (Li Hu Jian, 0.5%) since 25th July. The insecticide product is an important commercial formulation and used with recommended dosage (120–140 g/667 m^2^) for planthopper control in the local rice field.

Twenty blocks (4 rice seedlings in each block) were randomly selected and marked with plastic labels in the first and third rice fields. Thirty blocks (4 rice seedlings in each block) were randomly selected and marked with plastic labels in the second rice field. Population dynamics of the planthopper *N. lugens* were investigated every 5 days in each block of the three rice fields. The agronomic practices on the rice fields, such as fertilization and irrigation, followed the local recommendations commonly applied in the area where the experimental fields are located.

### 2.5 Data analysis

The Kolmogorov–Smirnov and Levene's tests were used to ensure that the data met the assumptions for parametric analyses (normal distribution of residuals and homogeneity of error variances). Survival rates were analyzed using the Kaplan–Meier method, and the log‐rank test was used to evaluate the significance of differences between two groups. One‐way ANOVA with *LSD* test (the least significant difference test) was used to analyze the predatory capacity and the number of *N. lugens* in the field experiment. All data was analyzed using SPSS 26.0.

## 3 RESULTS

### 3.1 Survival of *P. pseudoannulata*

**FIGURE 1:**
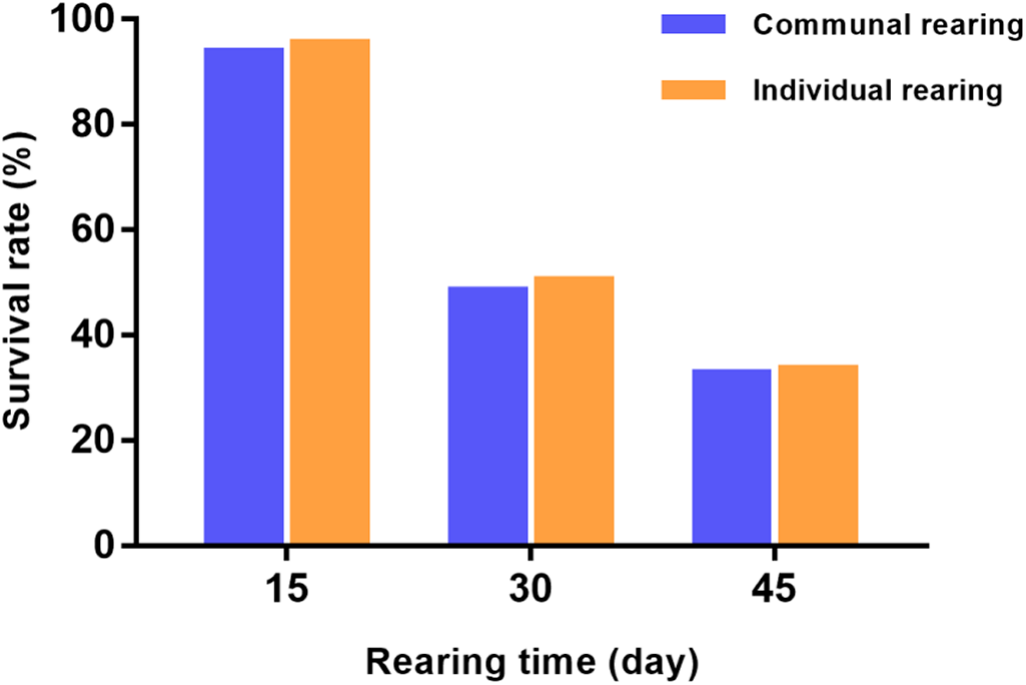
Survival rates of the communal‐reared and individual‐reared *Pardosa pseudoannulata* spiderlings after fifteen, thirty, and forty‐five days. Data represent the mean.

The survival rates of *P. pseudoannulata* spiderlings reared according to the two methods above are compared in Figure 1. At 15 days, both survival rates are above 95%, and the survival rates between two rearing group were not significantly different (*χ* ^*2*^ = 0.559, *p* = .455). Both groups' survival rates decreased to 50% at thirty days and fell below 40% at forty‐five days. No significant differences were found between the groups reared at thirty (*χ* ^*2*^ = 0.149, *p* = .699) or forty‐five days (*χ* ^*2*^ = 0.037, *p* = .847).

### 3.2 Predatory capacity

**FIGURE 2:**
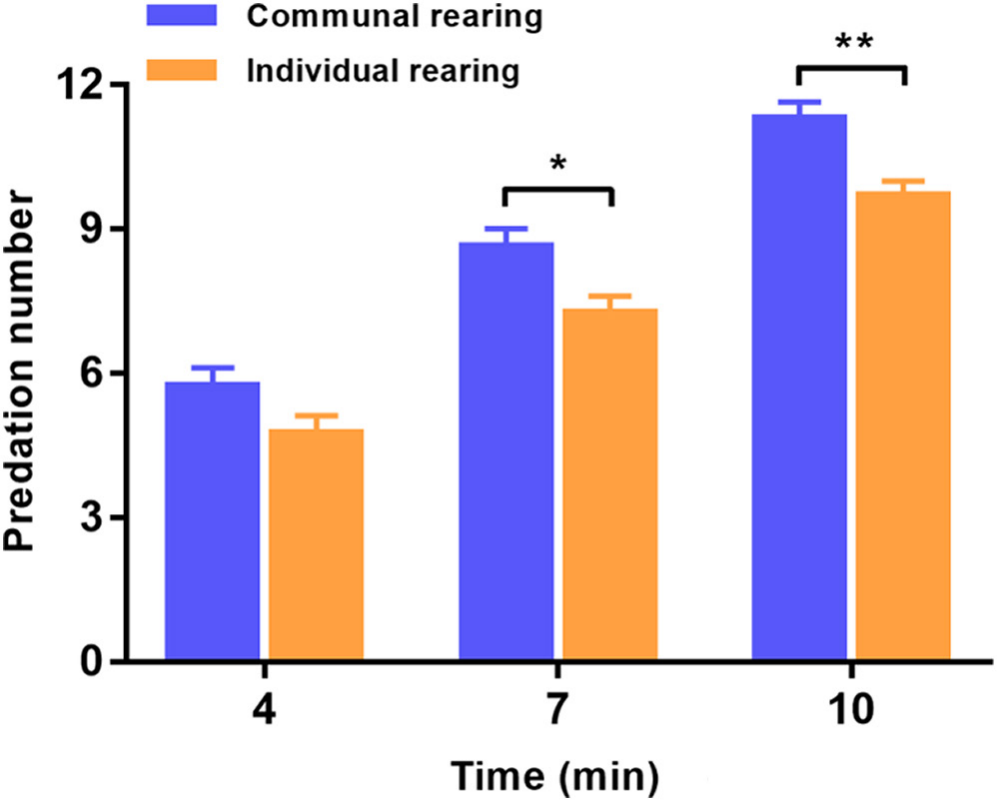
Predation capacity of *P. pseudoannulata* reared in two different methods after four, seven, and 10 min. Data represent mean ± standard error. One‐way ANOVA with *LSD* test was used to analyze the predatory capacity. An asterisk (*) denotes a significant difference between two rearing methods. *p < .05, **p < .01.

The predatory capacities of spiderlings from the two rearing methods are shown in Figure 2. At each time point, spiderlings reared together were able to hunt more fruit flies than those reared individually. Significant differences were detected between the two groups after hunting for seven (*F*_1, 58_ = 6.861, *p* = .011) and 10 min (*F*_1, 58_ = 12.197, *p* = .001). No significant difference was found after hunting for 4 min (*F*_1, 58_ = 3.288, *p* = .075).

### 3.3 Field experiment

**FIGURE 3:**
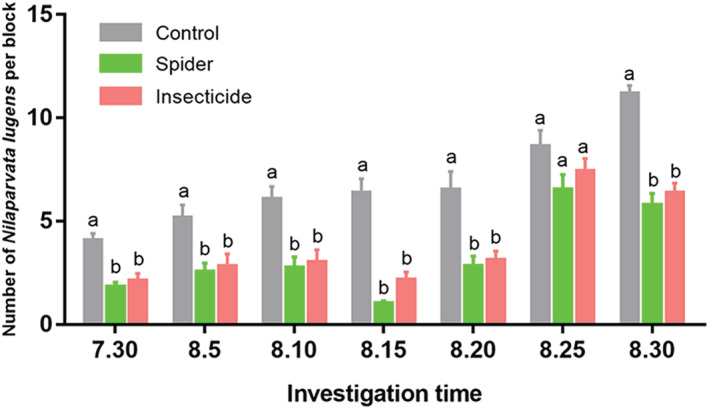
Population dynamics of the pest *Nilaparvata lugens* in rice fields under different treatments during August 2018 in the suburbs of Wuhan City. Control: no spider was released or insecticide was applied. “Spider” treatment: communal‐reared spiders were released on 25th July, and no insecticide was applied. “Insecticide” treatment: the rice field was applied with the botanical insecticide veratrine (Li Hu Jian, 0.5%). Data are represented as mean ± SE. One‐way ANOVA with *LSD* test was used to analyze the *N. lugens* number between treatments. Different lowercase letters indicate significant differences at *p* < .05.

The population dynamics of the pest *N. lugens* was investigated in the three rice fields were investigated, and the results were shown in Figure 3. Compared with the control, the spider treatment and insecticide treatment significantly reduced the *N. lugens* number (30th July: *F*_2, 67_ = 8.260, *p* = .001; 5th Aug: *F*_2, 67_ = 4.622, *p* = .013; 10th Aug: *F*_2, 67_ = 6.563, *p* = .002; 15th Aug: *F*_2, 67_ = 31.072, *p* < .001; 20th Aug: *F*_2, 67_ = 7.514, *p* = .001; 30th Aug: *F*_2, 67_ = 17.167, *p* < .001) except for 25th August (*F*_2, 67_ = 1.543, *p* = .221). No significant differences were found between spider treatment and insecticide treatment through all investigation date (30th July: *p* = .605; 5th Aug: *p* = .769; 10th Aug: *p* = .784; 15th Aug: *p* = .101; 20th Aug: *p* = .767; 25th Aug: *p* = .454; 30th Aug: *p* = .547).

## 4 DISCUSSION

The effects of early rearing experience on later foraging behavior of spiders have rarely been studied. In this study, we conducted a breeding study with the overall goal of addressing the development of co‐breeding protocols and testing whether these spiders are effective in biological control in field experiments. Survival rates and predatory capacity were determined and field experiment was conducted to investigate the population dynamic of *N. lugens* after release of communal‐reared spiders. The results showed that the survival of the two groups was close and communal‐reared spiders hunted more fruit flies than those reared individually. The number of the pest *N. lugens* was suppressed under the control of the communal‐reared spiders.

Survival rates in communal‐reared group and individual‐reared group were similar, but they dropped below 40% for both groups at forty‐five days. A possible explanation for this might be that the prey fruit flies did not provide sufficient nutrition, as *P. pseudoannulata* is a generalist predator. The prey species consumed by generalist predators could influence their growth, development, and survival (Toft & Wise, 1999). A mixture of prey is beneficial, because a broader range of nutrients can be obtained (Bernays et al., 1994; Heong et al., 1991; Raubenheimer et al., 2009; Uetz et al., 1992). Previous studies have reported that lycosid spiders often die before maturing when raised on only one prey type (Greenstone, 1979; Miyashita, 1968; Uetz et al., 1992). The performance of spiders fed a combination of prey (mixed diets) can be higher than that of spiders fed a monotypic diet (Wilder & Rypstra, 2008b). The fruit flies in our experiment were cultured in a medium composed mainly of corn meal, sucrose, and yeast extract powder, which lacks protein and lipid, both of which have been shown to be important for spider performance (Wilder, 2011). Another explanation for the low survival rates in the communal‐reared groups is that the spiderlings are cannibalistic. For solitary spiders, siblings of the same species are known to be cannibalistic in their interactions (Foelix, 2011). Previous research has reported that interclutch cannibalism is expected to occur much more frequently than intraclutch cannibalism (Iida, 2003). In our study, to avoid sibling cannibalism, the spiderlings in each bottle were from the same clutch. Cannibals usually prey on smaller conspecifics in order to avoid the risk of retaliation. Because cannibalism is most frequently a predator–prey interaction, its frequency should respond to the ecological factors of cannibal density, alternative prey (both abundance and relative food quality), and habitat structure, all of which can act separately or together to determine the frequencies of encounters with potential cannibals or potential prey (Wise, 2006). In our study, the prey (i.e. fruit flies) provided for the communal‐reared spiders were adequate, so the cannibalism only occurred at low rate in the communal‐reared spiders (based on laboratory observation).

Many studies demonstrate that spiders can adjust their behavior according to previous social experiences. Subadult females of the wolf spider *Schizocosa uetzi* that were exposed to mature males of a particular phenotype were subsequently more likely to mate with a male of a familiar phenotype as adults (Hebets, 2003). Furthermore, experience may also influence the courtship behavior and preferences of males of the wolf spider *Schizocosa ocreata*, as a study has shown that previous experience with female chemical cues can result in changes in courtship vigor (Moskalik & Uetz, 2011). In the wolf spider *S. ocreata*, penultimate females with more male visits are more selective as adults (Stoffer & Uetz, 2015), females exposed to only large‐tufted males or males with a mixture of tuft sizes demonstrated more receptivity displays to large‐tufted males than small tufted males (Stoffer & Uetz, 2016). In a burrow‐digging spider *Allocosa senex*, males with rejected experience enlarged their burrows more frequently and burrows were longer compared to non‐exposed males, indicating that males have plasticity in digging behavior in response to the availability of females (Carballo et al., 2017). In our study, plants were added to group rearing to simulate the social environment in which they lived in preparation for subsequent field experiments. The results showed that different rearing settings can influence the later foraging behavior of the solitary wolf spider. The *P. pseudoannulata* reared in groups were able to hunt more fruit flies than those reared individually. Similarly, a previous study showed that providing juvenile spiders with a larger container and a painted dowel significantly affected the behavior of spiders compared to those reared in a smaller, empty container (Carducci & Jakob, 2000). In our study, the spiders reared as a group had more space to move around, which was likely beneficial for the physical development of the spiders. Communal rearing may have made the spiders more competitive, as they had to move fast to reach the prey or otherwise starve.

Spiders are the most prominent generalist predators in rice fields and play a key role in controlling rice pests (Yang et al., 2018). Many species of spiders occupy diverse habitats in agroecosystems and prey upon a wide range of insect pests of various sizes, making them useful agents in biological control (Mansour et al., 1981; Riechert & Bishop, 1990). A total of 30 species of wolf spiders (Lycosidae), belonging to seven genera, were found in China rice fields, and *P. pseudoannulata* is a dominant species among these spiders (Yang et al., 2018). It has been reported that *P. pseudoannulata* can consume up to 12 rice planthoppers or leafhoppers per day (Wang, 2007). In practice, omnivory and cannibalization is the restriction factor of spider large‐scale breeding. Mixed diets and individual rearing require large amounts of human labor. Our results indicated that the survival of the single prey‐fed and communal‐reared spiders is similar to the individual‐reared spiders. In predatory capacity assays, the communal‐reared spiders hunted more preys than the individual‐reared spiders. Our results indicate that the wolf spider *P. pseudoannulata* could be reared at large‐scale and their predation capacity was augmented. It is worth noting that communal‐reared spiders could only be reared to around fourth instar, but the field experiment showed that these spiderlings had promising pest‐control effect.

The release of communal‐reared spider significantly reduces the population dynamics of the pest *N. lugens*, and the pest‐control effect is comparable with the insecticide. The larger container and rice plants in the communal rearing group may make the spider better predator. Our results suggest that spider release could suppress the damage of pests, and might reduce insecticide use. Evidence is growing that spiders can be effective biological control agents (Hodge, 1999). Increasing spider density in agroecosystems could open up the possibility of biological control of pest populations (Alderweireldt, 1994). Our results also has implications in practical applications, to some extent, it might be sustainable and cheaper to use lab‐reared spiders instead of insecticides in the future. Moreover, the synergistic or agonistic association between spiders and insecticides as pest control agents should be further explored.

In this study, we aim to develop a communal rearing protocol for spiders. We tested how the protocol affected the spider behavior compared to individually reared spiders that were reared under common laboratory conditions. We found that communal‐reared spiders were significantly more voracious. Since we changed more than one variable in the communal rearing protocol (i.e., spider density and environmental enrichment), it could not be determined which variable was responsible for the changes in spider behavior. Field experiment showed that release of communal‐reared spiders significantly suppressed the pest *N. lugens* population, and the pest‐control effect was similar to the insecticide. Our study provides reference for the large‐scale breeding of spiders and their use in the biological control.

## AUTHOR CONTRIBUTIONS

**Yaqi Peng:** Investigation (lead); writing – original draft (lead). **Fan Zhang:** Investigation (supporting). **Die Hu:** Investigation (supporting). **Dong Li:** Investigation (supporting). **Yao Zhao:** Supervision (lead); writing – review and editing (lead). **Yu Peng:** Project administration (lead); writing – review and editing (lead).

## CONFLICT OF INTEREST STATEMENT

The authors declare no conflict of interest.

## Supporting information


Figure S1.

## Data Availability

The data that support the findings of this study are available in Mendeley Data at https://data.mendeley.com/datasets/bz526drn49/2.
