# Supplementary material for: Communal rearing induces high predatory capacity in a solitary wolf spider and its potential in pest control
Source: Ecol Evol. 2023 Apr 18;13(4):e10024. doi: 10.1002/ece3.10024 (PMC10111170; doi:10.1002/ece3.10024)
Supplement: Supplementary file 1 — Figure S1. [file ECE3-13-e10024-s001.docx]

**
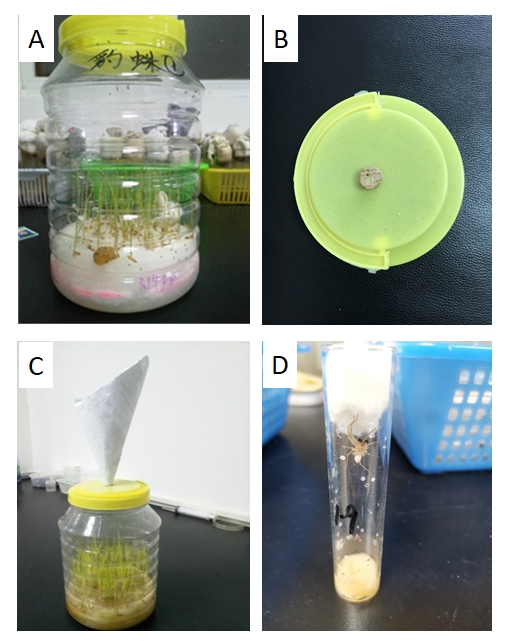
**

**Figure S1.** Two different methods of rearing the wolf spider *Pardosa pseudoannulata*. A, B, and C denote communal rearing, D denotes individual rearing. (A) Rearing bottle, (B) Bottle cap, (C) Funnel for fruit flies transport, (D) Individual rearing in glass tube.
